# Functional Characterization of *Solanum tuberosum* ER Lumen Binding Protein (*StBiP*) Genes Through Complementation in Yeast *kar2* Deletion Mutants

**DOI:** 10.3390/ijms27073094

**Published:** 2026-03-28

**Authors:** Binita Adhikari, Donna M. Gordon, Jeanmarie Verchot

**Affiliations:** 1 Department of Plant Pathology and Microbiology, Texas A&M University, College Station, TX 77845, USA; binita.adhikari@ag.tamu.edu; 2 Department of Biological Sciences, Mississippi State University, Mississippi State, MS 39762, USA; gordon@biology.msstate.edu

**Keywords:** *Saccharomyces cerevisiae*, heterologous expression, molecular chaperones, BiP, Kar2, unfolded protein response, membrane proteins, endoplasmic reticulum, protein folding

## Abstract

Yeast models are widely used to study molecular chaperones from diverse organisms, including plants, because of their well-characterized genetics and the conservation of the protein-folding machinery among eukaryotes. Cross-species complementation studies in yeast have yielded valuable insights into conserved biochemical activity and molecular functions that manage protein folding, assembly, and repair during stress. This study evaluated the functional capacity of three potato *StBiP* isoforms (*StBiP1*, *StBiP2*, and *StBiP3*) to complement the *kar2* deletion (*kar2Δ*) strain under a range of environmental and ER stress conditions. All three *StBiPs* partially restored colony growth under normal conditions, demonstrating that they are functional orthologs of yeast *KAR2* and can support core ER housekeeping functions. Under severe stress, however, the isoforms diverged: *StBiP3* most effectively complemented the *kar2Δ* strain during heat- and chemically induced ER stress, whereas *StBiP1* and *StBiP2* provided weaker protection. Unfolded protein response (UPR) activation, monitored via *HAC1* mRNA splicing, further highlighted isoform-specific differences in how the StBiPs support IRE1-HAC1 signaling under ER stress and oxidative stress. A conserved cysteine in the nucleotide-binding domain, previously implicated in Kar2 redox control, was also critical for StBiP3-mediated protection in yeast, although the same mutation led to different consequences in plant tissues. Together, these findings provide evidence of subfunctionalization among potato BiP isoforms, with StBiP3 emerging as a stress-specialized chaperone that is a promising target for improving ER stress resilience in solanaceous crops.

## 1. Introduction

The endoplasmic reticulum (ER) serves as a central hub for protein synthesis, quality control, and lipid biosynthesis. It also coordinates signaling with other organelles to support plants’ metabolic health and adaptation to environmental challenges. Misfolded proteins can either be degraded or refolded with the help of molecular chaperones. The immunoglobulin binding protein (BiP), also known as glucose-regulated protein 78 (GRP78) in mammals or Kar2 in yeast, is the principal facilitator of nascent protein folding in the endoplasmic reticulum (ER) and a central player in the unfolded protein response (UPR) [1,2,3,4]. Inositol-requiring enzyme (IRE1) is a key ER stress sensor that is kept inactive via BiP/GRP78/Kar2 binding to its luminal domains [5,6,7,8,9]. BiP depends upon ATP transporters to import high levels of ATP into the ER for chaperone-driven protein folding as well as IRE1 binding [10,11]. During ER stress, Kar2/BiP dissociates from IRE1, promoting IRE1 oligomerization, autophosphorylation, and cytosolic endoribonuclease activity. Activated IRE1 performs controlled splicing of an unconventional intron from the mRNAs of the yeast *HAC1*, mammalian *XBP1*, or plant *bZIP60* transcripts [8,12,13]. In each system, the translation of the spliced mRNA generates a transcription factor that stimulates expression of ER chaperones (including Kar2/BiP), providing a feedback loop to support protein folding and assembly.

BiP is one of the most abundant proteins in the ER. The crystal structures of yeast Kar2 and human BiP/GRP78 revealed that, like most HSP70 chaperones, both contain a nucleotide-binding domain (NBD) connected by a flexible loop region to a substrate-binding domain (SBD) [14,15]. BiPs recruit and fold substrates as they cycle through nucleotide binding and hydrolysis, a process aided by a nucleotide exchange factor (NEF) known as Sil1 [14]. Additionally, BiP substrate recognition and folding are accelerated by the aid of J-domain co-chaperones (ERdjs) [14,15,16]. Human BiP and yeast Kar2 share high sequence similarity, including with respect to ER targeting and retention motifs, while plant BiPs have distinct ER targeting and retention motifs. Since the late 1980s, scientists have known that human *BiP* can rescue *kar2* null or temperature-sensitive mutants, indicating that they share common chaperoning functions in the ER [1,2,17].

In contrast to yeast and mammals, plant *BiP* genes belong to a multigene family. *Arabidopsis thaliana*, citrus (*Citrus sinensis*), soybean (*Glycine max*), pepper (*Capsicum annuum* L.), tobacco (*Nicotiana tabacum*), rice (*Oryza sativa*), potato (*Solanum tuberosum*), and wheat (*Triticum aestivum*) each encode between three and six BiPs. Prior phylogenetic analysis has revealed that the plant BiP amino acid sequences are more distantly related to BiPs belonging to animals, yeast, and other organisms [18,19,20,21]. In Arabidopsis, soybean, and potato, *BiP1* and *BiP2* are broadly expressed, whereas *BiP3* is preferentially responsive to chemically induced ER stress and heat stress. Overexpression of selected Arabidopsis, pepper, soybean, potato, and tobacco *BiPs* enhances tolerance to drought and heat while modulating programmed cell death [21,22,23,24,25,26,27]. These patterns are increasingly viewed as evidence of subfunctionalization within plant *BiP* families [22,27,28,29]. To date, most evidence of the subfunctionalization of plant BiPs has come from analysis of cis-acting elements in *BiP* promoters that revealed the involvement of development or stress-responsive transcription factors. These studies were able to link specific environmental or developmental cues to different types of BiP gene expression [19,24,29]. By contrast, there is relatively little information on whether subfunctionalization also arises from structural or amino acid differences among BiP proteins themselves, raising the question of how functional diversification reflects regulatory versus protein-level divergence.

Baker’s yeast, *Saccharomyces cerevisiae*, has been a preferred eukaryotic system for studying protein folding and post-translational modifications, including glycosylation and disulfide-bond formation. It is also a preferred model for studying ER-stress survival, oxidative stress, and programmed cell death, with well-characterized pathways linking reactive oxygen species (ROS) to ER stress and cell fate. The extensive work performed on yeast to uncover how mammalian BiP/GRP78 functions at the mechanistic, residue, and pathway levels shows how yeast can be genetically manipulated in ways that are not feasible in mammalian cells or possible in plant cells. Although research tools that engage the yeast model for studying mammalian BiP/GRP78 have been developed, this system is underutilized for investigations of plant paralogs. In fact, only one study has reported yeast complementation by a tobacco *BiP* gene, primarily demonstrating complementation of a temperature-sensitive *kar2* mutant [30].

In order to investigate hypotheses concerning the subfunctionalization of plant BiP isoforms, we employed yeast as a heterologous system to study and functionally compare three potato BiP isoforms: StBiP1, StBiP2, and StBiP3. This study investigated whether each isoform could replace yeast Kar2 and how well they could sustain growth and UPR signaling under heat, chemical, and oxidative ER stress. We tested core chaperoning activities and stress protection potential to determine whether the potato BiP family behaves as a set of largely redundant ER chaperones or whether individual isoforms play specialized roles in maintaining proteostasis during severe stress.

## 2. Results

### 2.1. Structural Conservation of Solanum tuberosum BiP Proteins

**Figure 1:**
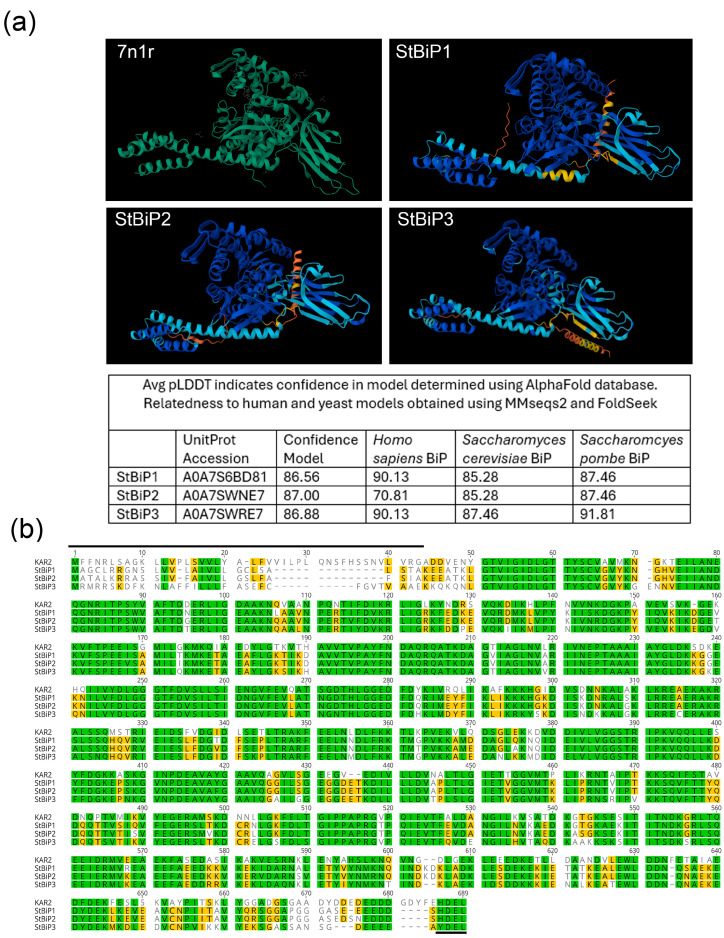
Structural models and sequence features of yeast Kar2 and potato StBiPs. (**a**) High-resolution (2.03 A) model of BiP (PDB: 7n1r) residues 25–633 and generated structures of the full-length StBiP1, StBiP2, and StBiP3, including N- and C-terminal signal peptides. AlphaFold-generated structures are colored according to pLDDT model confidence, with dark blue representing domains with exceedingly high confidence (pLDDT > 90), light blue indicating high confidence (pLDDT > 70), yellow indicating low confidence (pLDDT > 50), and orange denoting the lowest confidence (pLDDT < 50). The N-terminal signal sequences of the plant BiPs, which are absent in the PDB 7n1r model, are presented in yellow and orange. (**b**) Alignment of KAR2 and StBIP amino acid sequences shows significant homology. Black lines identify the N-terminal and C-terminal signal sequences. Identical amino acids are highlighted in green; functionally conserved amino acids are highlighted in yellow. The lines near the N-and C-termini highlight the ER transport and retention signals.

High-confidence models of the *Solanum tuberosum* BiP1, BiP2, and BiP3 proteins were generated using the AlphaFold 3.0 structural prediction tool (Avg pLDDT > 86%; Figure 1a). All StBiPs were found to exhibit high structural similarity with *Homo sapiens* (Avg pLDDT > 90), *S. cerevisiae* (Avg pLDDT > 85), and *S. pombe* BiP (Avg pLDDT > 87) using the MMseeks2 and Foldseek algorithms (Figure 1a). In monomeric unmodified BiP models (with no substrate or post-translational modifications), the nucleotide-binding (NBD) and substrate-binding (SBD) domains were highly conserved in overall folds and relative orientation relative to human and yeast BiPs, including the characteristic two-lobe organization of the NBD and the arrangement of α-helices and β-strands (Figure 1a). The structural lobes of the NBD contain surfaces corresponding to known co-chaperone and nucleotide-exchange factor (NEF) interaction sites described for yeast and mammalian BiPs [31,32,33]. Sequence analysis of the Sil1-interaction surface or the Sec63 J domain-interaction surface identified five Kar2/BiP residues (Arg217, Arg310, Glu311, Glu328, and Asp330) that are critical for these partnership interactions (Figure 1b). These residues and their local sequence contexts are highly conserved in StBiPs and Kar2. Arg217 lies in a sequence extending from positions 188 to 237 that shares complete identity between StBiPs and Kar2. Arg310 and Glu311 lie in a conserved KLKREAE sequence, with only a single Ala-to-Cys substitution in StBiP3. Glu328 and Asp330 are within a conserved RV(I)EID(E) sequence where the Asp330 position is occupied by Glu in the StBiP sequences [14,15,33,34]. Arg317 and Ala318, previously shown to modulate Sil1 binding to Kar2, are also conserved, except for StBiP1, which has an Ala-to-Ser substitution at position 318.

The SBDβ contains a polypeptide binding pocket and is connected by a short linker to the NBD. Its binding affinity for nascent peptides is allosterically coupled with the ATP-bound state of the NBD. Substitution mutations replacing Thr473 in Kar2 or in the human BiP, replacing only Thr229, or replacing the entire TAS(V)DNQP with a shortened 3-amino-acid sequence, are known to reduce polypeptide binding affinity in vitro, compromise the ATPase rate (allosteric coupling), and influence protein self-associations that can complicate crystallization [35]. The StBiPs maintain a conserved Thr473. The extended motif is TYQDQQT, and it is unknown whether this context influences substrate-binding affinity or molecular allostery. Other crucial amino acid positions that influence cold or heat tolerance are also conserved among StBiPs: A194, A203, A415, P464, and P515 [36].

### 2.2. Three Solanum tuberosum BiP Genes Partially Complement the Loss of KAR2 in Yeast

Global sequence alignment of the yeast Kar2, StBiP1, StBiP2, and StBiP3 proteins showed a high degree of sequence similarity, with only major differences occurring in the N-terminal signal peptides that mediate signal-recognition particle (SRP)-dependent targeting to the ER and the C-terminal ER-retention motifs. Kar2 has an N-terminal 42-amino acid signal peptide that is removed by proteolytic cleavage at G/ADD, with ADDVENY representing the first seven amino acid residues of the ER-lumenal protein [37]. The C-terminal ER-retention tetrapeptide is His-Asp-Glu-Leu (HDEL) for Kar2, StBip1, and StBiP2, whereas StBiP3 has Tyr-Asp-Glu-Leu (YDEL) (Figure 1b).

The *S. cerevisiae KAR2* is an essential gene, as haploid cells lacking a functional *KAR2* are inviable [2,38]. To evaluate whether potato BiPs can substitute for Kar2, we constructed plasmids in which *StBiP1*, *StBiP2*, and *StBiP3* coding sequences were placed under the control of the *KAR2* promoter, and their endogenous N-terminal signal peptides were replaced with the Kar2 signal sequence to ensure proper targeting in yeast. We fused the sequence encoding amino acids 1 to 50 ending with G/ADDVENY, which includes the G/A cleavage site plus amino acids that are specific to Kar2, to the downstream sequences, which in the alignment (Figure 1b) are identical from positions 51 to 60. A point mutation was introduced into *StBiP3* to convert its C-terminal YDEL to HDEL (Tyr → His) to match the canonical yeast ER-retention signal and promote efficient ER localization [1,4,39,40].

**Figure 2:**
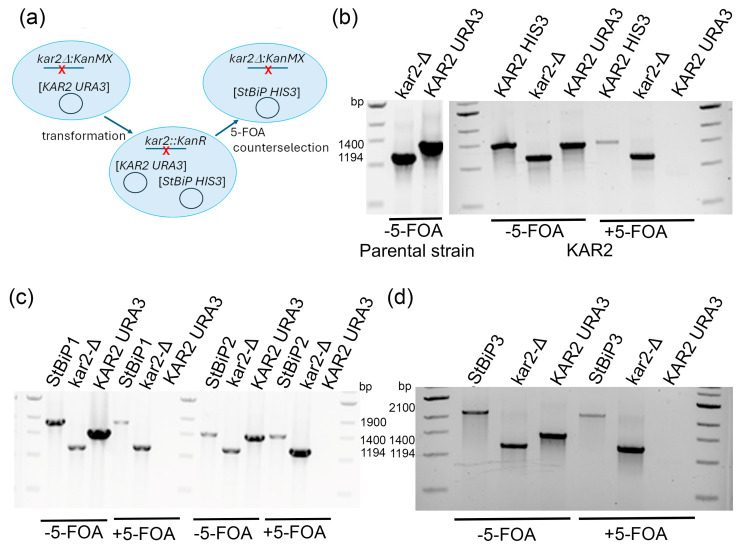
The plasmid-shuffling strategy and PCR verification of strain composition. (**a**) The schematic outlines the plasmid-shuffling strategy, in which 5-FOA counter-selection was used to remove the KAR2-URA3 cover plasmid prior to complementation testing. (**b**–**d**) PCR verification of strain composition before and after 5-FOA counterselection. (**b**) PCR verification of the *kar2*Δ*::KANMX* chromosomal deletion (1194 bp product) and plasmid-borne KAR2 (1400 bp product). The gel on the left shows parental strains maintained on non-selective media prior to transformation. The gel on the right shows PCR products from colonies immediately after transformation, which carry both KAR2-HIS3 and KAR2-URA3 plasmids, and colonies after 5-FOA selection, which retain only the *HIS3*-marked plasmid. (**c**,**d**) PCR verification of *HIS3*-marked plasmids before and after selection confirming the presence of KAR2/BiP constructs and loss of KAR2-URA3 cover plasmid. Gel images show 1900 bp, 1500 bp, or 2100 bp PCR products corresponding to StBiP1, StBIP2, or StBiP3. PCR verification of *kar2* deletion was performed.

Two haploid *kar2*Δ::*KanMX* yeast strains, DGY738 and DGY740, each containing a covering *URA3*-marked plasmid expressing the wild-type *KAR2* from the *KAR2* promoter, were used for complementation testing. Plasmid shuffling was performed by transforming each strain with *HIS3*-marked plasmids expressing *StBP1*, *StBIP2*, and *StBiP3* and then conducting 5-FOA counter selection to eliminate the KAR2-URA3 ‘cover’ plasmid, as detailed in the Section 4. This process yielded six independent transformant lines. An unmodified *HIS3*-marked vector, referred to as the ‘empty’ plasmid, served as a plasmid-shuffling control (Figure 2a,b). For each strain–plasmid combination, ten 5-FOA-resistant colonies were screened via PCR, with a 1194 bp product confirming insertion of *KanMX* at the endogenous *KAR2* locus. PCRs were performed immediately after transformation to identify colonies carrying both plasmids (KAR2-URA3 and each *HIS3*-marked plasmid) and again after 5-FOA selection to confirm the loss of the *URA3*-marked plasmid and retention of the *HIS3*-marked plasmids containing *KAR2*, *StBiP1*, *StBiP2*, or *StBiP3*. The PCR products indicating the KAR2-, StBiP1-, StBiP2-, and StBiP3-containing *HIS3*-marked plasmids were 1400, 1900, 1500, and 2100 bp in size, respectively (Figure 2b–d and Table S1). Three to four verified clones per combination were selected for further analysis.

**Figure 3:**
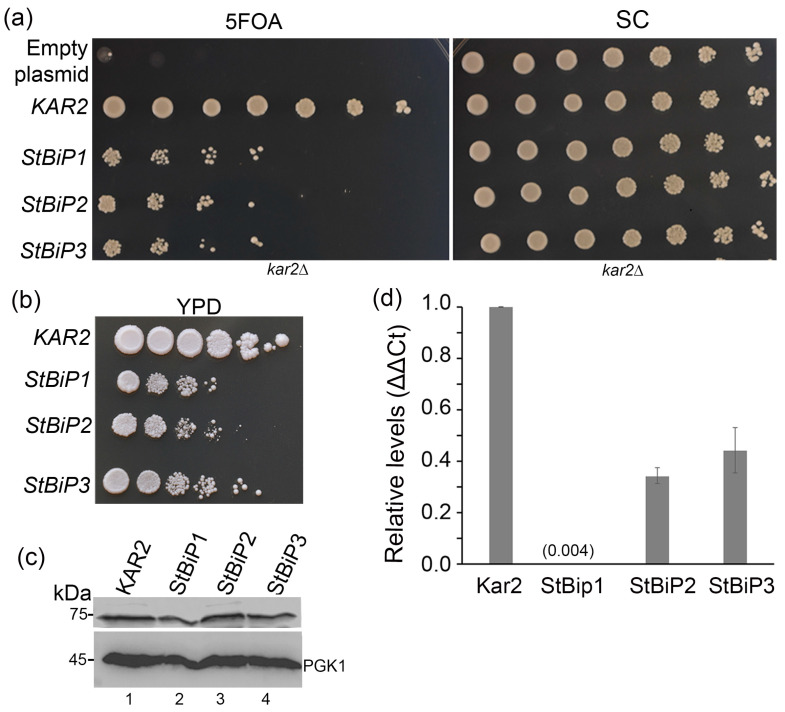
*KAR2* and *StBiP1*, *StBip2*, and *StBip3* complementation of *kar2*Δ yeast. Cultures were 5-fold serially diluted, spotted onto medium, and grown for 3–5 days at 30 °C. Growth comparison of serially diluted cells on (**a**) synthetic complete (SC) medium, with and without 5-fluoroorotic acid (5-FOA), or (**b**) on YPD medium following 5-FOA counterselection. Growth assays were spotted in duplicate and independently repeated three times. Representative images are shown. (**c**) Immunoblot detection of steady-state levels of Kar2p/StBiP proteins. PGK1 served as the loading control. (**d**) Relative mRNA transcript levels for episomal *KAR2* and *StBiP1*, *StBiP2*, and *StBiP3*, as determined by q-RT-PCR analysis. Average fold differences are reported relative to endogenous *KAR2* transcript levels for three independent experiments (using two or three replicates per genotype). ANOVA confirmed the samples were distinct from each other (*p* < 0.05). The level of StBiP1 is too low to show graphically and is represented by the value directly (0.004).

As an initial test of functionality, serial dilution assays were performed on 5-FOA selection medium using four colonies of each DGY738 and DGY740 transformant expressing *StBiP1*, *StBiP2*, or *StBiP3*. All StBiP proteins rescued the Δ*kar2* growth defect, although not to the same extent as Kar2p (representative data in Figure 3a). Partial restoration of growth defects was also observed on YPD agar (Figure 3b). To quantify differences in complementation, the average doubling time in liquid media was determined for three colonies of each DGY738 transformant. Kar2-expressing cells doubled in ~1.7 h, StBiP3 doubled in ~3.8 h, and StBiP2 doubled in ~5.6 h. StBiP1 cell growth was the most restricted over 24 h (Figure S1). Immunoblot analysis using a commercial antibody confirmed the presence of a ~75 kDa polypeptide for Kar2p, StBiP1, StBiP2, and StBiP3, a size that is consistent with ER targeting and signal sequence removal (Figure 3c) [38].

While *KAR2* and *StBiP* genes are expressed using the exact same promoter, their transcript levels may vary due to differences in mRNA stability or other post-promoter factors affecting RNA accumulation. To evaluate transcript levels, we used qRT-PCR to assess *KAR2* and *StBiP* transcript levels after 5-FOA counterselection and calculated *StBiP* abundance relative to plasmid-borne *KAR2*. Unexpectedly, *StBiP1*, *StBiP2*, and *StBiP3* transcript levels were lower than those for *KAR2*, even though all the genes were expressed from the same promoter and plasmid backbone (Figure 3d). *StBiP2* and *StBiP3* reached approximately 30 and 40% of the levels for *KAR2*, respectively, while *StBiP1* transcripts were barely detectable. The level of *StBiP1* was 75- to 100-fold lower than StBiP2 and StBiP3 (Figure 3d). Statistical analysis using ANOVA indicated that the transcripts for each KAR2/BiP isoform accumulated to distinct levels (*p* < 0.05). These results indicate that steady-state mRNA levels are not determined solely by promoter context [38].

### 2.3. Kar and StBiP Transformants Respond to Prolonged and Acute Heat Stress

**Figure 4:**
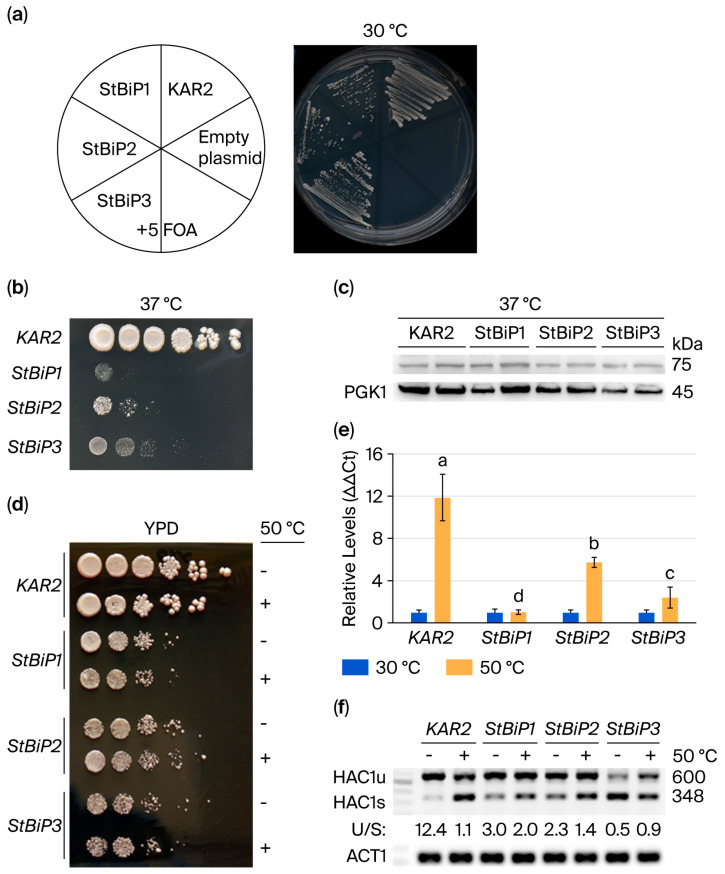
Cell growth following heat stress. (**a**) A streaked plate showing the growth of cells expressing Kar2 and StBiPs on 5-FOA medium under normal conditions. Liquid cultures were grown for the same time before using a loop to streak. (**b**) Ten-fold serial dilutions were spotted onto YPD medium and grown for 3 days at 37 °C. (**c**) Immunoblots using BiP sera and PGK1 sera (internal control) confirmed protein expression at 37 °C. (**d**) Untreated and heat-shocked liquid cultures (50 °C for 30 min) were 10-fold serially diluted and spotted onto YPD medium. A representative image of cells following 2–3 days of growth at normal temperatures is shown. (**e**) Relative transcript levels between normal and heat-shocked cultures determined by qRT-PCR with data normalized to untreated samples. Averages of three samples per genotype and treatment are shown. Experiments were repeated three times. Letters indicate statistical differences among samples as determined via ANOVA (*p* < 0.05). (**f**) RT-PCR was used to assess intron removal from *HAC1* mRNA.

Following 5-FOA selection and growth at 30 °C, cells were streaked onto a plate to reveal differences in growth (Figure 4a). Cells were also subjected to two separate heat stress regimes: (a) prolonged growth at moderately elevated temperatures, and (b) acute, high-temperature heat shock. For the prolonged-heat-stress assay, cells were serially diluted on YPD agar plates and incubated at 37 °C for 2 days (Figure 4b). Cells expressing wild-type Kar2p exhibited robust growth at 37 °C [41,42], whereas cells expressing *StBiP1*, *StBiP2*, or *StBiP3* showed reduced growth under these conditions (Figure 4b). Immunoblot analysis of biological replicates grown at 37 °C demonstrated that all strains had detectable levels of Kar2/StBiP proteins (Figure 4c). Together, these data indicate that potato BiPs only partially complement the heat-stress growth function of Kar2 despite being expressed at similar steady-state protein levels.

In a second acute thermosensitivity assay, exponentially growing cell cultures were exposed to 50 °C for 30 min and then spotted as serial dilutions onto YPD medium. For each strain, post-shock growth closely resembled that of the unstressed control, suggesting that acute 50 °C treatment did not further differentiate the growth of Kar2-, StBiP1-, StBiP2-, or StBiP3-expressing cells (Figure 4d).

In both yeast and plant cells, BiP family members (Kar2 in yeast and StBiPs in potato) regulate the ER stress sensor IRE1 by binding its ER lumenal domain and dissociating upon stress, thereby activating its endoribonuclease activity [17,43,44]. In yeast, IRE1 activation promotes unconventional splicing of *HAC1* mRNA, which, in turn, regulates *KAR2*/*BIP* transcription [45]. To monitor changes in *KAR2* and *StBIP* transcript levels following acute heat stress, we quantified mRNA levels using qRT-PCR. As expected, 50 °C heat shock induced a strong *KAR2* response, with the amount of *KAR2* transcripts increasing approximately 12-fold relative to the unstressed cells. In contrast, *StBiP1* transcript levels were unchanged, while the levels of *StBiP2* and *StBiP3* increased ~ 4-fold and 2-fold, respectively (Figure 4e). Statistical analysis demonstrated different transcript accumulation levels among *KAR2* and *StBiP*s at 50 °C (*p* < 0.05).

To assess induction of UPR, we examined *HAC1* mRNA splicing via semiquantitative RT-PCR using primers sitting in exons flanking the unconventional intron, yielding a 600 nt unspliced (HAC1u) and a 348 nt spliced (HAC1s) product (Figure 4f). Detecting two variants with a single primer pair provides an internal control for the assay. At the same time, data from an external reference gene (actin) ensures equivalent starting material between samples. A semi-quantitative approach avoids the differences in amplification efficiencies that may occur with using two primer pairs to detect the unspliced and spliced products [46,47,48,49]. Band densitometry was used to obtain a *HAC1u*:*HAC1s* ratio as an indicator for UPR signaling. The splicing ratio can also serve as a proxy for the effectiveness of Kar2 and StBiPs in restricting IRE1 endonuclease activity. In untreated cells, Kar2 was most effective at restricting *HAC1u* splicing, with a ratio of approximately 12:1. StBiP1 and StBiP2 were moderately restrictive, with ratios of approximately 3:1 and 2.3:1, respectively, whereas StBiP3 was the least restrictive and showed higher *HAC1s* accumulation. Following heat treatment, the KAR2 strain responded as expected, with the ratio of unspliced to spliced products shifting to approximately 1:1, which is consistent with robust IRE1 activation. The StBiP1, StBiP2, and StBiP3 strains showed only a mild increase in the spliced form, indicating partial heat-induced activation of IRE1. These data suggest that the StBiPs are comparatively inefficient at regulating IRE1 endonuclease activity (Figure 4f).

### 2.4. KAR2 and StBiP Transformants Respond to Chemically Induced ER Stress

**Figure 5:**
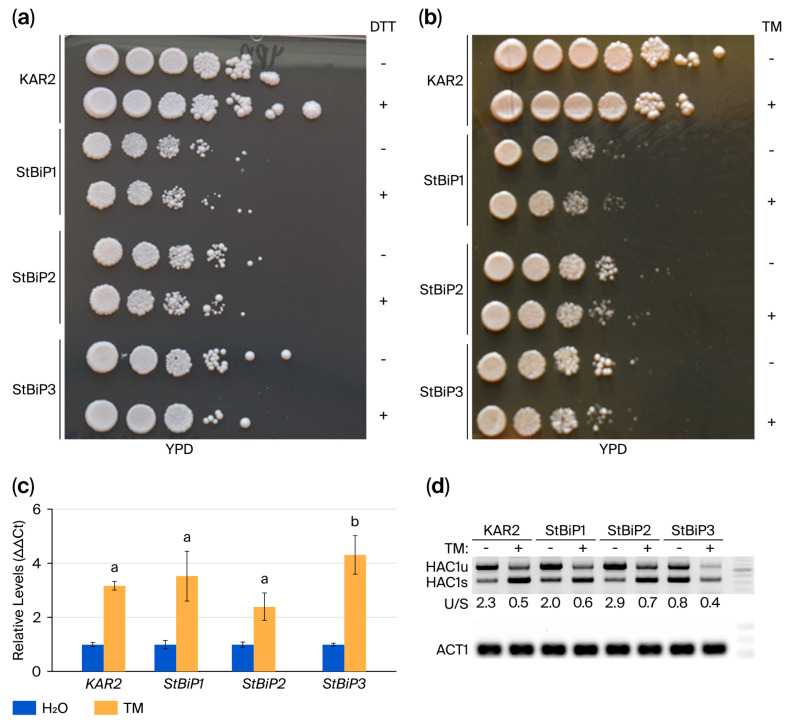
Cell growth following TM and DTT treatment. (**a**,**b**) Growth of cells following treatment with 5 µg/mL TM or 10 mM DTT (+) relative to the untreated controls (−). Spotting assay performed on YPD at 30 °C for 2–3 days. (**c**) qRT-PCR results showing *KAR2*, *StBiP1*, *StBiP2*, and *StBiP3* transcript levels after treatment with TM or water. The averages of three biological replicates for TM treatment were standardized to water-treated controls for each sample. The averages for the TM-treated samples were compared statistically (ANOVA), and only StBip3 yielded significant results. Letters above bars indicate statistical differences (*p* < 0.05). (**d**) Endpoint PCR showing accumulation of HAC1u and HAC1s following treatment with TM or water. Actin (ACT) primers were used as internal controls.

In budding yeast and plants, dithiothreitol (DTT) and tunicamycin (TM) are commonly used to induce ER stress and activate UPR. DTT can robustly activate UPR when applied at low concentrations (e.g., 2 mM), whereas higher concentrations, namely, 10 mM, can cause acute stress accompanied by loss of viability. TM at low doses, namely, 0.5 µg/mL, can initiate Kar-dependent ER stress protection, while higher doses, namely, 2.5 µg/mL, can cause a robust UPR accompanied by *KAR2* and *HAC1* induction [3,50,51,52,53,54]. In yeast and plants, TM-induced ER stress is accompanied by oxidative stress induced through increased lipid peroxidation, which also impairs cell growth and viability [55,56,57,58]. Here, cells were treated for 1 h with 10 mM DTT or 5 µg/mL TM, and serial dilutions were spotted onto YPD medium (Figure 5a,b). As expected, overall growth for the *KAR2*- and *StBiP1*-, *StBiP2*-, and *StBiP3*-expressing cells was comparable between the chemically treated and untreated controls (Figure 5a,b).

The *KAR2* promoter maintains gene expression at basal “housekeeping” levels under unstressed conditions and contains a motif known as the unfolded protein response element (UPRE) that is recognized by HAC1, a key transcriptional activator of UPR target genes, including *KAR2*. Given that HAC1 is responsive to TM treatment and the genes of interest are expressed from the same *KAR2* promoter, qRT-PCR was performed to assess KAR2/BIP induction. We found that the relative levels of *KAR2*, *StBiP1*, and *StBiP2* mRNAs were consistently elevated by approximately 2.5-fold compared to the untreated controls, whereas *StBiP3* showed a higher level of induction: 4.5-fold (Figure 5c). The *StBiP3* strain may have experienced higher ER stress under TM treatment than the other strains, causing greater upregulation of UPR.

In yeast, UPR signaling is responsible for unconventional splicing of HAC1 mRNAs. Semi-quantitative RT-PCR is often used to evaluate splice variants. We evaluated the ratios of *HAC1u:HAC1s* before and after TM treatment, using a semiquantitative RT-PCR assay and band densitometry (Figure 5d), expecting to see a shift in the ratio reflecting higher *HAC1s* accumulation. *KAR2* cells grown under normal conditions had an initial ratio of *HAC1u:HAC1s* of 2.3, but following TM-induced UPR, the ratio was 0.5, reflecting the increased proportion of *HAC1s* (Figure 5d). Similarly, the ratios of *HAC1u:HAC1s* were 2.0 for *StBiP1* and 2.9 for *StBiP2* cells grown under normal conditions. Following TM treatment, the ratios changed to 0.6 and 0.7, respectively. By contrast, the *StBiP3* cells presented a ratio of 0.8 under normal conditions, while the ratio was 0.4 following TM treatment (Figure 5d). Taken together, these data indicate that StBiP3-expressing cells exhibit higher *HAC1s* levels both before and after TM treatment and a less pronounced shift in *HAC1u:HAC1s* ratio compared to Kar2, StBiP1, or StBiP2, suggesting altered regulation of UPR signaling in the StBiP3 strain.

### 2.5. Growth of KAR2- and StBiP-Expressing Cells Following Oxidative Stress

Direct treatment of budding yeast cells with hydrogen peroxide (H_2_O_2_) has been used to probe links between oxidative stress and HAC1-dependent UPR activation, although the degree of UPR activation in this case is reported to be weaker than that caused by classical ER stressors such as TM [42,59,60]. In Arabidopsis, treatment with TM or H_2_O_2_ induces NADPH-oxidase-dependent reactive oxygen species (ROS) signaling that is associated with UPR activation, although it is unknown whether the ability of plant BiP homologues to protect cell viability or modulate UPR activation can be distinguished [61,62]. In plants, analysis of the oxidative stress protection provided by individual StBiP isoforms is challenging because StBiP1-3 encodes highly similar proteins, making loss-of-function mutations in a single gene difficult to evaluate. Only differences between their promoters have allowed plant BiP3s to be identified as more ER-stress-induced and likely contributors to protection during episodes of oxidative stress [25,26]. Thus, yeast offers a tractable system with which to explore isoform-specific roles using separate complementation lines.

**Figure 6:**
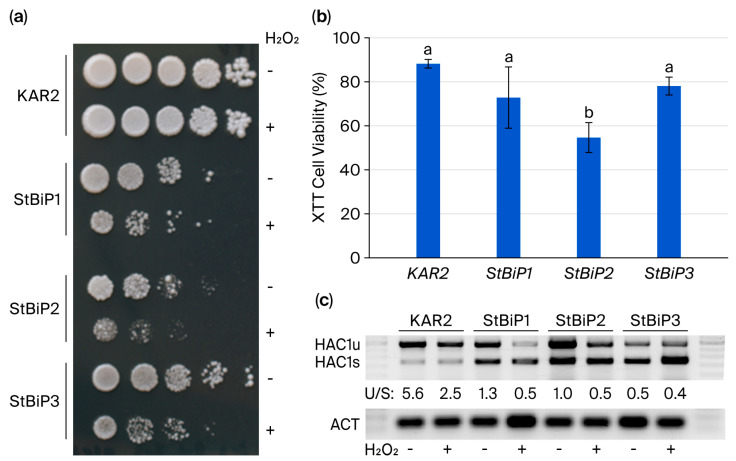
Kar2- and StBiP-expressing cells treated with hydrogen peroxide. (**a**) Exponentially growing cultures were treated with water (−) or 4 mM H_2_O_2_ (+), 10-fold serially diluted, and spotted on YPD medium. A representative photo depicting growth following 2–3 days at 30 °C is shown. (**b**) Quantitative determination of cell viability following H_2_O_2_ exposure. Cell viability was determined spectrophotometrically (490 nm) using the XTT assay. Data are reported relative to untreated controls. Averages were determined from 3 independent experiments. Letters above bars indicate statistical differences (ANOVA; *p* < 0.05). (**c**) Endpoint PCR shows accumulation of *HAC1u* and *HAC1s* following treatment. *Actin* (ACT) primers were used as internal controls.

To compare how *KAR2*- and *StBiP*-expressing yeasts respond to peroxide-mediated stress, exponentially growing cultures were treated with 4 mM H_2_O_2_ for 30 min and then spotted as serial dilutions onto YPD medium. Under these conditions, peroxide treatment did not detectably affect the growth of *KAR2*-expressing cells, whereas StBiP1, StBiP2, and StBiP3 strains were negatively impacted, with the StBiP2 strain appearing the most sensitive (Figure 6a). To quantify this effect, a colorimetric XTT cell viability assay was carried out, revealing an approximately 10–20% reduction in the number of viable KAR2, StBiP1, and StBiP3 cells and an over 40% loss of viability among StBiP2 cells. Overall, the percentages of metabolically active *KAR2*, *StBiP1*, and *StBiP3* cells were statistically similar, whereas *StBiP2*-expressing cells were significantly more susceptible to peroxide toxicity (Figure 6b; *p* < 0.05). For all strains, peroxide treatment resulted in comparable changes in *HAC1u:HAC1s* ratios (Figure 6c), indicating that peroxide exposure was sufficient to activate IRE1. Thus, the differences in H_2_O_2_-dependent loss of cell viability indicate that StBiPs are less efficient at protecting cells against such stress than Kar2.

**Figure 7:**
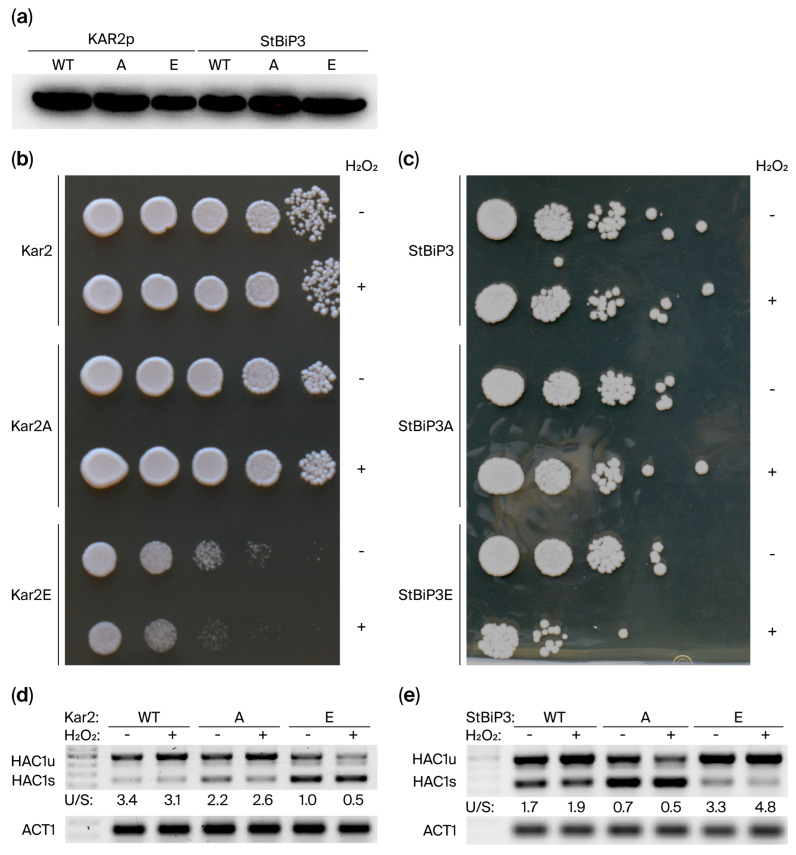
Cys63 mutants play diverse roles in oxidative stress protection during hydrogen peroxide treatment. (**a**) Immunoblot confirming the expression of the wild-type and modified Kar2 and BiP3 proteins. (**b**,**c**) Spotting assay using 10-fold serially diluted cultures pretreated with water (−) or 4 mM H_2_O_2_ (+). Representative photo shown for growth on YPD medium following 2–3 days at 30 °C. (**d**,**e**) Endpoint RT-PCR showing relative accumulation of *HAC1u* and *HAC1s* mRNAs following exposure to hydrogen peroxide. *Actin* (ACT) primers were used as internal controls.

Kar2 contains a conserved cysteine (Cys63) in its ATPase domain that can be directly modified by oxidants, which can decouple the ATPase and peptide-binding activities, thereby altering the Kar2 chaperoning mechanism. Previous work has already shown that replacing Cys63 with Ala has no effect on cell viability and does not alter chaperoning activities or the UPR in cells treated with heat or DTT [41,42,62]. In contrast, replacing Cys63 with a negatively charged residue such as Glu was reported to decrease cell viability and elevate UPRE-driven gene expression under normal and heat-stressed conditions [41,42]. We generated Cys63 to Ala or Glu substitutions in both Kar2 and StBiP3 to determine whether the conserved ATPase domain Cys is required for ER stress adaptation and cell viability during peroxide stress [41,60]. Immunoblot analysis confirmed expression of the wild-type and mutant Kar2 and StBiP3 proteins under normal growth conditions (Figure 7a).

Cells were treated with 4 mM H_2_O_2_ for 30 min, and then serial dilutions were spotted onto YPD medium. The peroxide-treated and untreated KAR2 and KAR2-Cys63A strains grew similarly, indicating that Ala had no effect, as expected. The KAR2-Cys63E strain showed reduced growth under no-stress conditions, with a further reduction in growth following peroxide treatment (Figure 7b,c), which is consistent with a previous report [41]. In contrast, StBiP3, StBiP3-Cys63A, and StBiP3-Cys63E strains grew similarly under no-stress conditions. These data suggest that the Glu substitution is less deleterious in StBiP3 than in Kar2, where Cys63E impairs viability. Following peroxide treatment, StBiP3-Cys63E growth was reduced, suggesting that the similar Cys63 residue in StBiP3 is also important for oxidative-stress protection. Together these results indicate that while the conserved Cys63 confers oxidative stress protection in both Kar2 and StBiP3, the Cys63E substitution is likely more detrimental in Kar2 than in StBip3.

Since *HAC1s* is the active (spliced) form, we used the ratio of *HAC1u/HAC1s* as a proxy for UPR activation in no-stress and peroxide-treated cells (Figure 7d,e). For both the KAR2 and KAR2-Cys63A strains, this ratio was unchanged between no-stress and peroxide-treated conditions, suggesting that peroxide treatment did not significantly trigger the UPR in these backgrounds. Surprisingly, the Cys63E mutation impacted Kar2 and StBiP3 differently. Whereas the KAR2-Cys63E mutant displayed HAC1 processing with increased *HAC1u/HAC1s* ratios following peroxide treatment, very little if any HAC1 was processed in the StBiP3-Cys63E mutant in no-stress and peroxide-treated conditions (Figure 7d,e). These data suggest that, for both Kar2 and StBiP3, Cys63 is important for proper management of UPR under oxidative ER stress. Thus, the conserved Cys63 residue in Kar2 and StBiP3 appears to link oxidative stress protection with regulation of *HAC1* mRNA splicing as an indicator of UPR signaling.

### 2.6. StBiP3 Cys63 Protects Against Oxidative Stress in Plant Tissues

**Figure 8:**
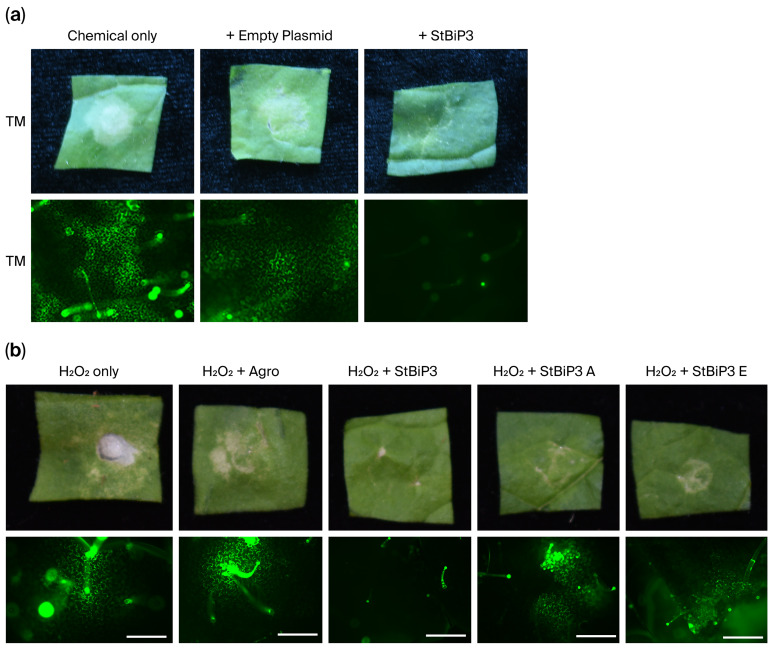
Transient expression of *StBiP3* confers protection against ER stress in plant tissues. Leaf segments were photographed under the normal light setting following 2 h of treatment. Epifluorescence images taken following H_2_DCFDA staining to detect ROS production. (**a**) *N. benthamiana* leaf segments treated with 5 µg/mL tunicamycin (TM). Images show the effects of agrobacterium alone or agro-delivered StBiP3 on leaf necrosis. (**b**) Necrosis in agro-infiltrated or control leaves treated with 4 mM H_2_O_2_. H_2_DCFDA staining was used to detect ROS production following peroxide treatment. Transient expression of *StBIP3* conferred protection, while Cys63A and Cys63E mutants were susceptible. The white scalebar represents 300 µm.

Yeast has a single ER stress response governed by IRE1-HAC1, while plants have multiple and partially redundant pathway branches (IRE1-bZIP60 and bZIP28 branches), complicating genetic analysis. Given that yeast and plants share similar core ER chaperone and redox machinery, we examined whether insights gained using yeast can help address questions that are difficult to resolve in planta. Guided by the data obtained using yeast, we overexpressed StBiP3 in *Nicotiana benthamiana* leaves via Agrobacterium infiltration. Leaf segments were treated with TM followed by a vital dye, H_2_DCFDA, which becomes fluorescent when oxidized by reactive oxygen and programmed cell death (Figure 8a). Here, TM treatment alone caused substantial tissue necrosis and H_2_DCFDA fluorescence, the scale of which was alleviated by StBIP3 overexpression. Similarly, tissues treated with peroxide showed necrosis and strong fluorescence, the scale of which was reduced by StBIP3 overexpression (Figure 8b).

Given the role of the residue Cys63 in Kar2 and BiP3 regarding ER-redox protection in yeast, we next expressed *StBIP3-Cys63A* or *StBiP3-Cys63E* in *N. benthamiana* leaves and treated tissues with peroxide. Substituting Cys63 with Ala or Glu compromised ER stress protection, resulting in increased tissue necrosis and H_2_DCFDA fluorescence (Figure 8b). These data indicate that StBiP3 can confer protection against ER-redox stress in both yeast and plant cells and that its conserved Cys63 residue is required for cytoprotective activity.

## 3. Discussion

Because plants have high genetic redundancy and expansive BiP families, a single gene deletion does not always lead to a clear phenotype. This can make it difficult to assign specific molecular functions to individual BiP paralogs. By expressing plant BiPs in yeast, we were able to bypass redundancy and directly test how StBiPs individually contribute to ER stress responses. Under normal conditions, the three StBiP proteins partially supported the growth and colony formation of a *kar2*-deficient yeast strain, demonstrating that StBiPs can carry out the general housekeeping functions of Kar2. This experimental system will enable future investigation into how individual protein domains or amino acids contribute to ER chaperone activity and stress responses.

Despite their high sequence homology, *StBiP* genes show distinct expression patterns across developmental stages, tissue types, environmental conditions, and ER stress levels, indicating strong transcriptional regulation rather than major divergence in core chaperone functions [18,22,24,27,63]. Promoter swap experiments support this idea, as BiP proteins are often interchangeable when expressed from the same promoter within a given species [20,24]. Building on this framework, we expressed three *Solanum tuberosum* BiPs from centromeric plasmids in a *kar2*-deficient yeast background, under the control of a native *KAR2* promoter, to focus our investigations on their intrinsic chaperoning activities and stress-protective functions. The *KAR2* promoter contains independent heat shock (HSE) and UPR elements that act additively to maximize induction [64] and allow endogenous-like control of these constructs.

Quantitative RT-PCR analysis revealed that steady-state StBiP mRNA levels were lower than those of the plasmid-based *KAR2*, although BiP protein was present for all three StBiPs. This observation points to possible intrinsic differences in mRNA stability or feedback control of protein expression. Future work will focus on discriminating between these possibilities. Regarding the endpoint RT-PCR and densitometry, which provided a semi-quantitative readout of *HAC1* splicing and changes in the *HAC1u:HAC1s* ratio, the changes in principle arise from alterations in total *HAC1* transcription as well as IRE1 endonuclease activity. In this study, we interpreted the shifts in *HAC1u:HAC1s* ratio in the context of IRE1 activity, as all strains were analyzed side-by-side under identical conditions and the direction of change was consistent across multiple treatments and biological replicates. Nevertheless, this study does not distinguish between altered splicing efficiency and potential changes in overall *HAC1* transcription. Further in-depth work will provide insight into whether StBIPs under various stresses influence IRE1 endonuclease activity, changes in HAC1 transcription, or a combination of both.

Moreover, we used HAC1u/HAC1s mRNA ratios as a proxy for IRE1 activity and UPR signaling throughout this study. While HAC1 splicing is a necessary and conserved feature of the UPR, it does not directly measure the efficiency of interactions between StBiP1, StBiP2, or StBiP3 and IRE1. In fact, in plants, very little is known about how different BiPs physically interact with the IRE1 lumenal domain in vivo or whether plant IRE1 isoforms (e.g., Arabidopsis IRE1a, IRE1b, and IRE1c) can functionally substitute for yeast IRE1. Our data are consistent with the concept of individual BiPs suppressing IRE1 endonuclease activity to different degrees, but they do not directly establish the underlying binding or kinetic mechanisms. To address this issue, future work will require direct testing of BiP-IRE1 interactions, cross-species complementation of IRE1 in yeast, and quantitative HAC1 as well as plant bZIP60 splicing assays.

Structural modeling and amino acid sequence comparisons revealed that the StBiP1, StBiP2, and StBiP3 investigated closely resembled yeast and human BiPs, supporting the notion that StBiPs can perform conserved ER chaperone functions across kingdoms, including yeast strains lacking functional Kar2. The highly conserved residues implicated in Sil1 and Sec63 interaction suggest that StBiPs are likely to engage these co-chaperones and support BiP ATPase cycling, although this remains to be directly tested. Previous studies with Kar2 mutants have explained how specific regions of BiP interact with Sil1 and other ER DnaJ co-chaperones (Sec63p, Jem1p, and Scj1p), providing a framework for future investigations of StBiP behavior in yeast [2,34,65,66].

The partial complementation of *kar2Δ* suggests there are functional differences in chaperone activities between the StBiPs and Kar2, and these differences become more apparent under thermal stress. Longer-term growth at 37 °C, which requires transcriptional reprogramming and metabolic adjustment, was better supported by StBiP2 and StBiP3 than by StBiP1. In contrast, acute heat shock at 50 °C, which triggers rapid induction of the activity of heat shock proteins and chaperones to prevent protein aggregation, resulted in similar colony growth among the StBiP strains, suggesting that all three isoforms can support core protein-folding functions to manage acute proteotoxic stress [67,68,69].

During acute chemical stress caused by DTT or TM, Kar2 chaperoning functions are preserved, and colony growth is protected [67]. Under these conditions, all three StBiP strains showed similar degrees of colony growth, indicating that they can substitute for Kar2 in maintaining cell viability. StBiP1 and StBiP2 showed *HAC1u:HAC1s* ratios similar to those of Kar2, suggesting comparable control of IRE1 activation. StBiP3 showed poorer apparent regulation of IRE1 under no-stress conditions but produced a robust HAC1 splicing response to TM, indicating its participation in UPR activation under strong ER stress.

Oxidative stress caused by peroxide treatment in yeast can activate HAC1 signaling and trigger redox signaling for cell survival [45,70]. The StBiP2 strain showed the most obvious reduction in colony density and cell viability following peroxide treatment relative to the untreated controls and the KAR2 strain, indicating that StBiP2 provides weaker protection against oxidative ER stress. The extent of *HAC1u* mRNA splicing increased after peroxide treatment in KAR2, StBiP1, and StBiP2, but not detectably in StBiP3, an outcome similar to what was observed during acute heat stress. These data highlight isoform-specific differences in how StBiPs support UPR signaling.

To better understand the differences between StBiP3 and Kar2, we focused on a conserved redox-active cysteine that is present in the nucleotide-binding domain (Cys63 in Kar2) [42,60]. Substituting Ala for Cys63 in the KAR2 and StBiP3 strains had no obvious effect on essential chaperoning activities that support colony growth, whereas the Glu mutation did not have the same effect on StBIP3 growth as on KAR2 cells. Differences were also seen regarding HAC1u splicing. These differences suggest that the conserved Cys and surrounding residues shape the domain conformation, and we can only speculate that this might influence interactions with cochaperones or other factors.

In conclusion, this study demonstrates that yeast is an effective model for dissecting the multiple biological roles of plant BiPs in relation to similar roles of Kar2. We present evidence that the three StBiP proteins can substitute for essential Kar2 functions, despite differing in their ability to manage acute and chronic heat stress, chemical ER stress, and oxidative stress. Building on extensive molecular analysis of Kar2, our data indicate conserved post-translational control of BiP/Kar2 at Cys63 is a determinant of ER redox protection and UPR tuning. This study also revealed isoform-specific behavior in StBiP3 that is not apparent from sequence conservation alone. Considering that plants encode three or more BiPs, it is worth clarifying whether the expansion of this gene family in plants favors competition between BIP chaperone activities and UPR activation, like Kar2, or whether another regulatory model exists. Future work using the yeast system and complementary plant assays should clarify how individual StBiPs integrate chaperone activity, redox sensing, and UPR signaling for environmental and ER stress resilience.

## 4. Materials and Methods

### 4.1. Amino Acid Sequence Alignments and AlphaFold Protein Structure Prediction

Geneious Prime (version 2026.0.2) was used to perform MUSCLE alignments using sequences obtained from NCBI (MN982518.1, MN982519.1, MN982520.1, and YJL034W). The EMBL-EBI AlphaFold Protein Structure Database was used to compare structural conservation among BiPs (https://alphafold.ebi.ac.uk/; accessed on 15 August 2025) [71,72,73].

### 4.2. Construction of Saccharomyces cerevisiae kar2Δ Shuffle Strain

Cross-species complementation of plant *BiP*s in yeast was performed using two haploid *kar2* shuffle strains, which were generated for this study using well-established technologies [74]. Homologous recombination was used to replace one copy of the *KAR2* open reading frame (ORF) in the BY4743 diploid *S. cerevisiae* strain (MATa/α *ura3Δ0/ura3Δ0 his3Δ1/his3Δ1 leu2Δ0/leu2Δ0 LYS2/lys2Δ0 MET15/met15Δ0 KAR2/kar2*Δ::*KanMX*) with a modified *Neomycin phosphotransferase II* gene (KanMX6) as outlined by Longtine and colleagues [75]. The *kar2* deletion was verified through genomic PCR utilizing primers targeting sequences within the KanMX cassette and the *KAR2* promoter (Table S1). A *URA3* covering plasmid that contained the *KAR2* promoter-*KAR2* ORF (pDG413) was introduced into the heterozygous diploid Kar2 yeast strain via lithium acetate transformation to generate DGY724.

DGY724 was induced to undergo sporulation on a medium containing 1.5% potassium acetate, 0.05% glucose, 0.25% yeast extract, 2% agar, and the appropriate supplements [76]. After 5 days at 30 °C, ascospores were treated with Zymolyase^®^ 100T and micro-dissected on YPD medium. Only tetrads producing four viable spores were analyzed. Segregation of heterozygous alleles (2:2 pattern) was confirmed on -Met, -Lys, and YPD with 0.2 g/L G418 medium. The mating type (MATa or MATα) was determined through complementation tests with mating tester strains, and the presence of the pDG413 plasmid was confirmed via growth on -Ura medium. The pDG413 plasmid contains the entire *KAR2* promoter and ORF in the pRS416 backbone (Supporting Information Table S2) and was generated using intermediate plasmids pDG407 and pDG410, which contain the *KAR2pro* and *KAR2* ORF, respectively, in the pMINIT-2.0 vector backbone (New England Biolabs, Ipswich, MA, USA). Two haploid products from two different 4-spore tetrads, DGY738 (MATa) and DGY740 (MATα), were used for StBiP testing.

Yeast strains were kept under standard growth conditions at 30 °C in YPD medium (1% yeast extract, 2% peptone, and 2% glucose) or in a synthetic complete medium (SC, 0.67% yeast nitrogen base without amino acids, 2% glucose) with the appropriate supplements to ensure plasmid retention.

### 4.3. Construction of Test Plasmids Containing KAR2-Promoter-Driven StBiP or KAR2 ORFs

Details on plasmid construction and selectable markers are provided in Table S2. The pDG415 plasmid (*KAR2* promoter-*KAR2* ORF cassette with *HIS3*-selectable marker) was prepared by ligating a SalI-BamHI fragment from the pDG413 plasmid into the SalI-BamHI sites of the pRS413 plasmid [77].

The pBA1 series of intermediate plasmids contains the *KAR2* promoter and ORFs for *StBiP1*, *StBiP2*, or *StBiP3* (NCBI Gene IDs: MN982518.1, MN982519.1, and MN982520.1). The *KAR2* promoter sequence was PCR-amplified and introduced into the XhoI-linearized pRS413 plasmid [77] via homologous recombination using the In-Fusion Snap Assembly kit (Takara Bio USA Inc., San Jose, CA, USA). The *StBiP* ORFs were amplified via RT-PCR using total RNA extracted from *Solanum tuberosum* leaves (cultivar Russet Norkotah). RNA was prepared using the RNeasy Plant Mini Kit (Qiagen, Germantown, MD, USA). The Maxima™ reverse-transcriptase kit (Thermo Scientific, Waltham, MA, USA) was used to prepare cDNA. The first PCR amplification was performed using primer pairs with 15 nt additional sequences for ligation with the pBA1.0 backbone (Table S1).

The pBA2 series of constructs was developed using IN-FUSION^®^ technology to replace the 5’ signal sequences of each *StBIP* ORF with the 147 bp sequence encoding the Kar2 signal peptide. An HIS codon was introduced to replace an endogenous Tyr to improve ER retention of StBiP3. In the pBA3 plasmid series, substitution mutations were introduced into the *KAR2* or *StBIP* ORFs to replace cysteine 63 with alanine (A) or glutamic acid (E). PCR-amplified DNA fragments and linearized plasmids were ligated (Table S2). Plasmid sequences were confirmed via Sanger sequencing (Eton Biosciences, San Diego, CA, USA).

All test plasmids were individually transformed into the *kar2Δ* haploid shuffle strain using the Frozen-EZ Transformation II kit (Zymo Research, Irvine, CA, USA) with the transformants selected on -Ura -His medium. Counterselection was performed using -His +5-FOA medium [74]. Successful loss of the *URA3*-marked plasmid was confirmed by the absence of cell growth after individual colonies were streaked onto -Ura media. PCR amplification (primers in Table S1) was used to confirm the *kar2*Δ mutation and the presence/absence of the covering plasmid before and after 5-FOA selection.

Two haploid kar2Δ::KanMX yeast strains, DGY738 and DGY740, each containing a covering *URA3*-marked plasmid expressing the wild-type *KAR2* from the *KAR2* promoter, were used for complementation testing. Plasmids were shuffled by transforming each strain with *HIS3*-marked plasmids expressing *StBP1*, *StBIP2*, and *StBiP3* and then conducting 5-FOA counter selection to eliminate the KAR2-URA3 ‘cover’ plasmid. For each strain–plasmid combination, ten 5-FOA-resistant colonies were screened via PCR using a KanMX forward primer and a 3′ KAR2 UTR reverse primer, which generated a 1194 bp product confirming insertion at the *KAR2* locus. Stable presence of the KAR2-URA3 plasmid was confirmed via PCR using CovF and CovR primers, which generated a 1400 bp product (Table S1). Stable presence of each KAR2-, StBiP1-, StBiP2-, and StBiP3-containing an HIS3-marked plasmid was confirmed using M13F together with KAR2, StBiP1R, StBiP2R, or StBiP3R primers, which generated 1400, 1900, 1500, and 2100 bp products, respectively (Table S1). PCRs were performed immediately after transformation to identify colonies carrying both plasmids (KAR2-URA3- and *HIS3*-marked plasmids) and again after 5-FOA selection to confirm the loss of the *URA3*-marked plasmid and retention of the *HIS3*-marked plasmids containing *KAR2*, *StBiP1*, *StBiP2*, or *StBiP3*. At each step of transformation and selection, deletion of the endogenous *KAR2* was also confirmed. Three to four verified clones per combination were selected for further analysis.

### 4.4. Complementation Growth Assay

Transformed cells were grown overnight in -His medium. A cell-volume equivalent to 0.5 OD_600_ (~1 × 10^7^ cells) was 5-fold serially diluted in growth medium, and 3 µL of each dilution was spotted on 5-FOA-containing plates to remove the covering plasmid. The plates were photographed following incubation for 2–3 days at 30 °C.

### 4.5. Heat, Chemical, and Oxidative Stress Assays

Cells from an overnight liquid culture were diluted in fresh media to achieve a cell density of 0.1 OD_600_ and then grown for 3–4 h to reach 0.4–0.5 OD_600_. Cells were collected via centrifugation and resuspended in YPD medium to an OD_600_ of 0.5. The conditions used to induce ER stress or oxidative stress for spotting assays, qRT-PCR, and endpoint PC assays were consistently applied: cells were exposed to 10 mM of dithiothreitol (DTT) or 0.5 µg/mL tunicamycin (TM) for 2 h. Control cultures were treated with equivalent volumes of water (for DTT) or DMSO (for TM). For oxidative stress, cultures were divided and treated with 4 mM hydrogen peroxide for 30 min, as described by Tran and Green [78]. Cells from 1 mL samples were pelleted, washed with phosphate buffer (pH 7.5), and resuspended in 1 mL of water to attain 1.5 × 10^7^ cells/mL. Suspensions were serially diluted 10-fold, and 5 µL of each dilution was spotted onto YPD plates. Photos were taken at 48 h of growth at 30 °C. To determine cell viability, peroxide-treated and control cells were pelleted and resuspended in 1 mL phosphate-buffer saline (PBS). Cells were incubated for 2, 4, and 20 h with XTT reagent (Biotium Corp., Freemont, CA, USA) according to the manufacturer’s protocol. Absorbance data at OD = 490 nm were collected. The averages across all time points and across three experiments were statistically analyzed using ANOVA in JMP Student edition software (version 19.0.5).

### 4.6. Endpoint and qRT-PCR Analysis of Gene Expression

Yeast cells were cultured at 30 °C in YPD medium and harvested in the early logarithmic growth phase (OD_600_ ≤ 0.4). Cell volumes equivalent to 2.0 OD_600_ were collected via centrifugation, and total RNA was extracted by using the Maxwell RSC SimplyRNA Tissue Kit (Promega, Madison, WI, USA) according to the manufacturer’s protocol. Unspliced and spliced *HAC1* cDNAs were amplified via PCR using intron-flanking primers (HAC1F/HAC1R), producing a 600 nt unspliced product and a 348 nt spliced product (intron size is 252 nt). To ensure the quality and quantity of the cDNA used in PCR reactions, primers Act1F and Act1R were used to amplify a fragment of *ACT1* (Supporting Information: Table S1). Complementary DNA (cDNA) was synthesized from 100 ng of total RNA using a High-Capacity cDNA Reverse Transcription Kit (Applied Biosystems, Foster City, CA, USA) with random primers for cDNA synthesis. PCR was performed using a hot start to delay DNA polymerase activity by heating to 95 °C for 2 min and then employing 40 cycles of 95 °C for 15 s, 57 °C for 15 s, and 72 °C for 30 s, followed by 72 °C for 5 min and then chilling the product to 4 °C. ImageLab 6.1 software associated with the Bio-Rad Gel Doc imager was used for band densitometry (Bio-Rad Laboratories, Inc., Hercules, CA, USA).

qRT-PCR analysis of *StBiP* and *KAR2* gene expression was performed using 1 µL of cDNA, 900 nM primers, and the Power SYBR™ Green PCR master mix (Table S1). To assess the amplification efficiencies for each primer pair, a series of five 10-fold dilutions of cDNA were prepared and then used to perform qPCR. Standard curves were generated and the calculated efficiencies ranged between 90 and 100%. A QuantStudio™ 3 Real-Time PCR machine (Applied Biosystems) and the ThermoFisher Connect Platform were used for data analysis. A hot start delaying activation of the DNA polymerase (50 °C for 2 min and 95 °C for 2 min) was followed by 40 cycles of 95 °C for 15 s, 57 °C for 15 s, and 72 °C for 1 min. The next cycle was 95 °C for 15 s, followed by 55 °C for 1 min, and the final cycle was 95 °C for 15 s, 55 °C for 15 s, and 72 °C for 2 min. The relative level of RNA was calculated using the 2-ΔΔCT method. ACT1F and ACT1R primers were used to amplify *ACTIN* as an internal control to measure ΔCt. Where indicated, the endogenous KAR2 Ct or StBiP1 was used as the normalization control to calculate the ΔΔCt values. For the heat and Tm treatment samples, the ‘before treatment’ Ct values were used as an external control for calculating ΔΔCt. Each treatment was assessed with three technical replicates, and experiments were repeated three times (three biological replicates) for consistency. Statistical analysis (ANOVA) was performed using JMP Student edition software.

### 4.7. Immunoblot Analysis

Total cellular proteins were extracted from 1.0 OD_600_ cell equivalents using alkaline lysis followed by trichloroacetic acid (TCA) precipitation, as described in [79]. Protein samples were heated at 55 °C for 5 min, resolved via electrophoresis on a 10% SDS-PAGE gel, and transferred to a 0.4 μm PVDF membrane. We used anti-GRP78 sera (RRID: AB_2039169, Enzo Life Sciences, Farmingdale, NY, USA) and anti-PGK1 sera (RRID: AB_3695731, ProSci Inc., Poway, CA, USA) at dilutions recommended by the manufacturers, followed by HRP-conjugated secondary anti-rabbit sera (RRID: AB_430833, Promega, Madison, WI, USA). Protein detection was performed using the Clarity Max Western ECL Substrate, and digital images were captured using a ChemiDoc Imaging System (Bio-Rad, Hercules, CA, USA).

### 4.8. Construction and Testing of Binary Plasmids in N. benthamiana Leaves

IN-FUSION^®^ technology was used to generate the plasmid pCB301-StBiP3 and mutant versions containing Cys-to-Ala or Cys-to-Glu substitution mutations. Wild-type and modified StBiP3 ORFs were obtained via PCR amplification and ligated with the linearized pCB301 vector. Plasmids were transformed into the electrocompetent *Agrobacterium tumefaciens* strain GV3101 [47]. Overnight A. tumefaciens cultures were resuspended in infiltration buffer with 100 mM acetosyringone to 0.5 OD_600_ [47]. The cultures were incubated for 3–4 h in the dark before being injected into the underside of *N. benthamiana* leaves with a syringe. The plants were maintained for 2 days, and then the same leaves were injected with solutions of 10 mM DTT, 5 µg/mL TM, or 4 mM hydrogen peroxide. After 4 h, leaf segments were treated with 50 mM H_2_DCFDA dye and examined using an epifluorescence microscope.

## Data Availability

All data are available from the OAKTRUST repository at TAMU.

## Supplementary Materials

The following supporting information can be downloaded at https://www.mdpi.com/article/10.3390/ijms27073094/s1.

## Abbreviations

The following abbreviations are used in this manuscript:

BiP	Immunoglobulin-binding protein
GRP78	Glucose-regulated protein 78
ER	Endoplasmic reticulum
UPR	Unfolded protein response
IRE1	Inositol-requiring enzyme
NBD	Nucleotide-binding domain
SBD	Substrate-binding domain
NEF	Nucleotide exchange factor
ERdj	J-domain co-chaperones of ER
