# Supplementary material for: Functional Characterization of Solanum tuberosum ER Lumen Binding Protein (StBiP) Genes Through Complementation in Yeast kar2 Deletion Mutants
Source: Int J Mol Sci. 2026 Mar 28;27(7):3094. doi: 10.3390/ijms27073094 (PMC13072888; doi:10.3390/ijms27073094)

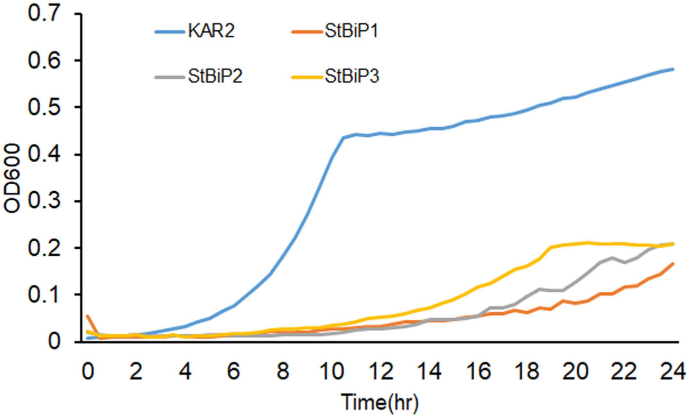

Figure S1: Growth curve representing two independent experiments. Liquid growth characteristics of StBiP-expressing yeast strains. Cells from overnight cultures were diluted to 0.01 OD600, and three technical replicates were added to 96 96-well plate to analyze growth using a SpectraMax iD5 plate reader. The OD600 reading was taken every 30 minutes over 24 hours. The X-axis represents the time, and the Y-axis represents the OD600 reading.

Table S1. Primers used in this study		
**Name**	**Primer sequence**	Notes
**Construction of pDG413 plasmid**		
DG336	GCGGTCGACTACCGGTGTCTCATCGTGGTCAAGAG	KAR2 promoter + 5' ORF amplification
DG337	CTTGAAAGCTTTTATCAATTGACGAACG	
DG338	GATAAAAGCTTTCAAGAAGAAGCATGG	KAR2 ORF amplification
DG339	GCGCTCGAGGGATCCCTACAATTCGTCGTGTTCGAAATAATC	
**Construction of *HIS*-marked pGD415 and BA1 test plasmids**		
DG375	ATTGGGTACCGGGCCCCCCCTACCGGTGTCTCATCGTG	KAR2 promoter amplification
DG376	GCTTATCGATACCGTCGACCGGTATGTTTGATACGCTTTTTC	
RS413BIP1F	CGTATCAAACATACCATGGCTGGCTGTTTGAGGC	StBiP1 first round amplification
RS413BIP1R	ATCGATACCGTCGACTTACAGCTCATCATGGCTGTCATC	
RS413BIP2F	CGTATCAAACATACCATGGCTACTGCGTTGAAGAGAC	StBiP2 first round amplification
RS413BIP2R	ATCGATACCGTCGACTTACAGCTCATCATGTGAATCATCG	
RS413BIP3F	CGTATCAAACATACCATGAGAATGAGACGTTCAAAGGA	StBiP3 first round amplification
RS413BIP3R	ATCGATACCGTCGACCTATAATTCATCATAAGCCTCCTCT	
DESTF	GTCGACGGTATCGATAAGCTTGA	Second round amplification and cloning
DESTR	GGTATGTTTGATACGCTTTTTCCCT	
**Replacing SRP to generate pBA2 expression plasmids**		
BIP123SRP-KF	ATCAAACATACCATGTTTTTCAACAGACTAAGCGCTGGC	First round amplification of 147 bp KAR2 signal coding sequence with ends overlapping StBiP genes
BIP1SRP-KR	TCCTATAACGGTACCGTAGTTTTCTACATCATCGGCACC	
BIP2SRP-KR	GCCAATAACGGTTCCGTAGTTTTCTACATCATCGGCACC	
BIP3SRP-KR	CCAATCACTGTTCCATAGTTTTCTACATCATCGGCACC	
BIP1SRP-KDEST F	GGTACCGTTATAGGAATTGACC	Destination vector amplification for introducing y 147 bp encoding KAR2 SRP
BIP2SRP-KDEST F	GGAACCGTTATTGGCATAGATC	
BIP3SRP-KDEST F	TGGAACAGTGATTGGAATTGATC	
BIP123 SRP-KDEST R	CATGGTATGTTTGATACGCTTTTTC	

**Primers used to generate BiP modifications and mutants**		
BIP3Y-H F	GATGAATTATAGGTCGACGGTATC	
BIP3Y-H R	GACCTATAATTCATCGTGAGCCTCCTCTTCCTC ATCTC	Tyr-His (ER retention)
BIP3(C-A)F	ATATTCTGCCGTGGGTGTATACAAAGGTGAGA AC	
BIP3(C-A)R	CCCACGGCAGAATATGTTGTCCCTAGATCAATT	Cys 63 to Ala
BIP3(C-E)F	ATATTCTGAAGTGGGTGTATACAAAGGTGAGA AC	
BIP3(C-E)R	CCCACTTCAGAATATGTTGTCCCTAGATCAATT	Cys63 to Glu
**Endpoint PCR for confirming plasmid shuffle**		
KANMX forward	GATTGTATGGGAAGCCCGAT	kar2Δ (1194bp)
3' KAR2 UTR reverse	CGAGCCTTTCAACTCTCTCT	
CovF	AGACGCATTGGGTCAACAGT	p(KAR2,URA3) (1400bp)
CovR	GAAAAGGGCGTACAGGACCA	
M13F	GTAAAACGACGGCCAGT	p(KAR2, HIS3) (1400bp)
KAR2 R	ACTTAGCTCTGGTCAAGGTTTC	
M13F	GTAAAACGACGGCCAGT	StBiP1 (1900bp)
StBiP1-R	GGGACTTCTTGGTTGGGATAA	
M13F	GTAAAACGACGGCCAGT	StBiP2 (1500bp)
StBiP2-R	GCCTTCTTGACAGGAGTCATT	
M13F	GTAAAACGACGGCCAGT	StBiP3 (2100bp)
StBiP3-R	GAGCATCCACCTTCTCCTTTAC	
**Endpoint RT-PCR and qPCR analysis of gene expression**		
HAC1F	TCGCACTCGTCGTCTGATA	
HAC1R	TCATGAAGTGATGAAGAAATCATTCACT	
Act1F	GGTTGCTGCTTTGGTTATTGA	
Act1R	TTTTGACCCATACCGACCAT	
StBiP1-F	GAGGGTGGAGACGAAACTAAAG	qPCR BiP1
StBiP1-R	GGGACTTCTTGGTTGGGATAA	
StBiP2-F	CTCTCTTCGATGGTGTGGATTT	qPCR BiP2
StBiP2-R	GCCTTCTTGACAGGAGTCATT	
StBiP3-F	CTAGTGACAAGTCGCGTCTAA	qPCR BiP3
StBiP3-R	GAGCATCCACCTTCTCCTTTAC	
Kar2qF	TCTGAAGGTGTCTGCCACAG	qPCR KAR2
Kar2qR	TTAGTGATGGTGATAGATTCGGATT	
**Cloning Kar2 mutant**		
KAR2-A For	TTATTCCGCCGTTGCTGTGATGAAAAATGGT	

**Table S2. Plasmids used in this study**					
Plasmid name	backbone	Insert	Restriction or In-Fusion® cloning	Cloning or Modifying Oligonucleotides	Selectable marker
pDG413	pRS416	KAR2pro:KAR2	SalI-BamHI		URA3
pDG407	pMINIT-2.0	KAR2pro	SalI-HindIII	DG336,DG337	
pDG410	pMINIT-2.0	KAR2 ORF	HindIII-BamHI	DG338, DG339	
pDG415	pRS413	KAR2pro:KAR2	SalI-BamHI		HIS3
pBA1.0	pRS413	KAR2pro	In-Fusion	DG375, DG376	HIS3
pBA1-StBip1	pBA1.0	StBiP1	In-Fusion	RS413BIP1F<br>RS413BIP1R	HIS3
pBA1-StBip2	pBA1.0	StBiP2	In-Fusion	RS413BIP2F<br>RS413BIP2R	HIS3
pBA1-StBip3	pBA1.0	StBiP3	In-Fusion	RS413BIP3F<br>RS413BIP3R	HIS3
pBA2.0-StBiP1	pBA1.0-StBiP1	KAR2 5' signal	In-Fusion	BIP123SRP-KF<br>BIP1SRP-KR<br>BIP1SRP-KDESTF<br>BIP1SRP-KDESTR	HIS3
pBA2.0-StBiP2	pBA1.0-StBiP2	KAR2 5' signal	In-Fusion	BIP 123SRP-KF<br>BIP2SRP-KR<br>BIP2SRP-KDESTF<br>BIP2SRP-KDESTR	HIS3
pBA2.0-StBiP3	pBA1.0-StBiP3	KAR2 5' signal<br>KAR2 3' signal	In-Fusion	BIP123SRP-KF<br>BIP3SRP-KR<br>BIP3SRP-KDESTF<br>BIP3SRP-KDESTR<br>BIP3Y-H F<br>BIP3Y-H R	HIS3
pBA3-KAR2C63A	pDG415	Substitution Cys63 with Ala	In-Fusion	KAR2A-F<br>KAR2A-R	HIS3
pBA3-KAR2C63E	pDG415	Substitution Cys63 with Glu	In-Fusion	KAR2E-F<br>KAR2E-R	HIS3
pBA3-BiP3C63A	pBA2.0-StBiP3	Substitution Cys63 with Ala	In-Fusion	BIP3(C-A)F<br>BIP3(C-A)R	HIS3
pBA3-BiP3C63E	pBA2.0-StBiP3	Substitution Cys63 with Glu	In-Fusion	BIP3(C-E)F<br>BIP3(C-E)R	HIS3

Original gels for Figure 2

panel (b)

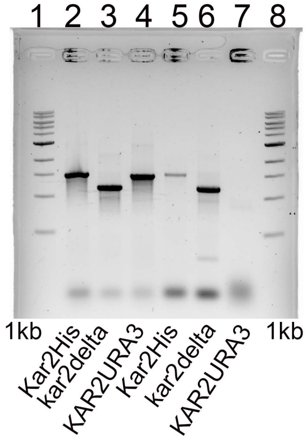

lanes 1, 8: 1 kb ladder
Lanes 2-4 is without 5-FOA
Lanes 5-7 is +5-FOA

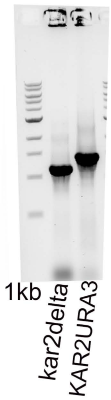

-5FOA
parental
strain

panel (c)

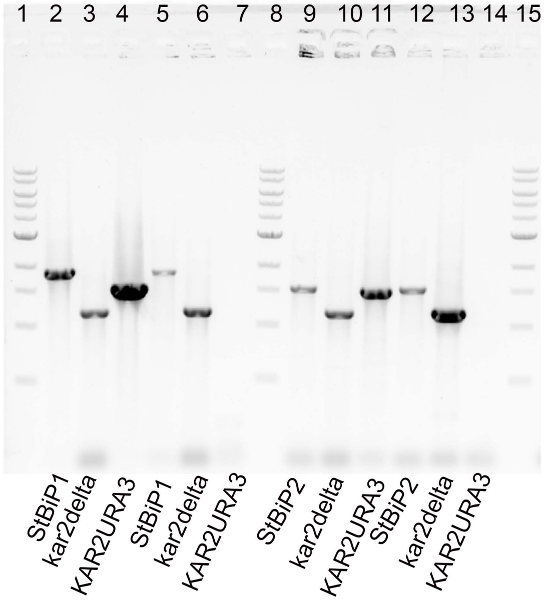

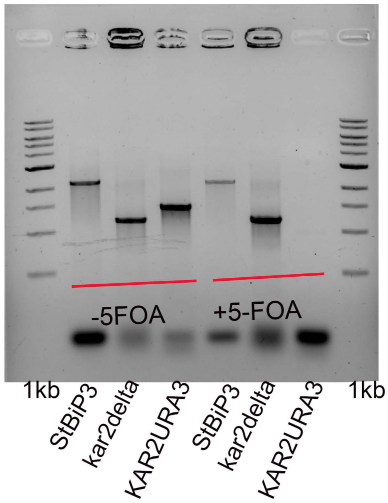

Figure 3 panel (c) western blot. Two antisera used at same time detecting 75 kDa BiP/Kar2 and 45 kDa PGK1.

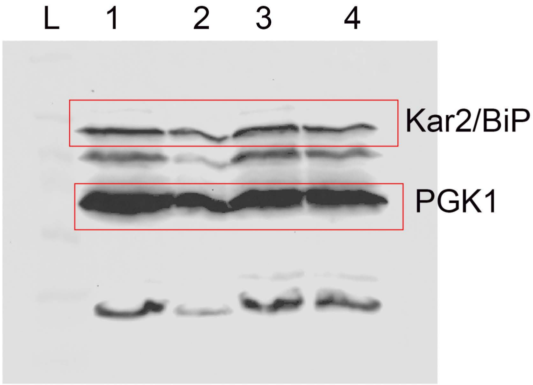

Figure 4 original gels

panel (c) western blots

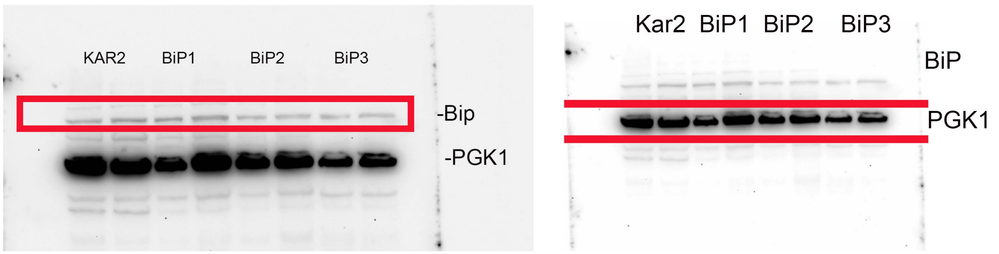

two exposures of the same gel. Selected bands highlighted in red to compile figure

panel (f) ethidium bromide stained gels

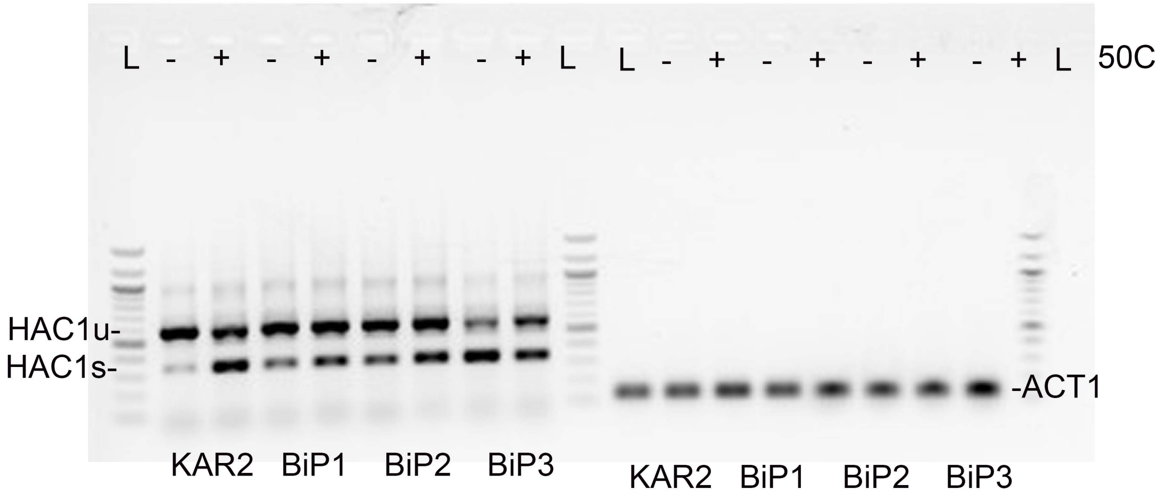

Figure 5 original panel (d)

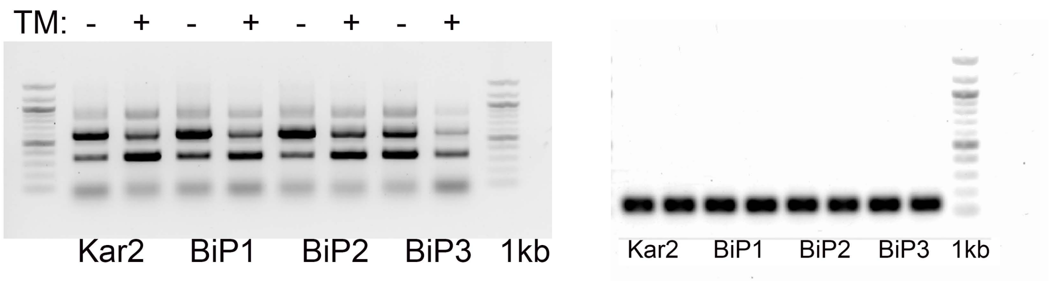

Original images for FIgure 6 panel (c)

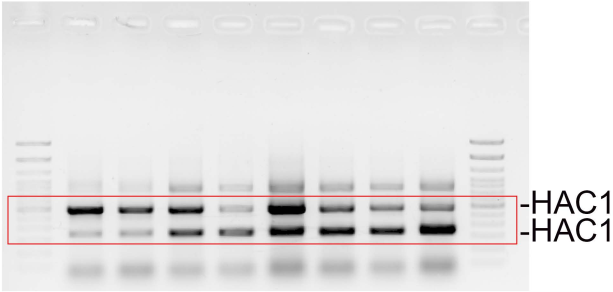

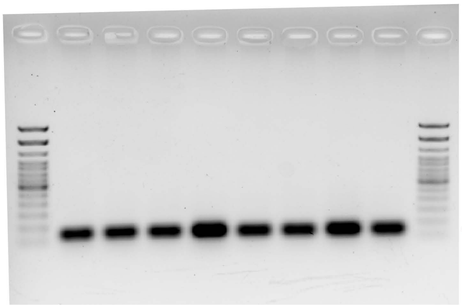

Original image for Figure 7 panel (a)

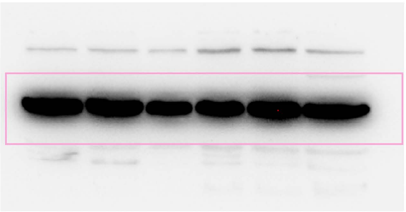

Oiriginal image for panel (d)

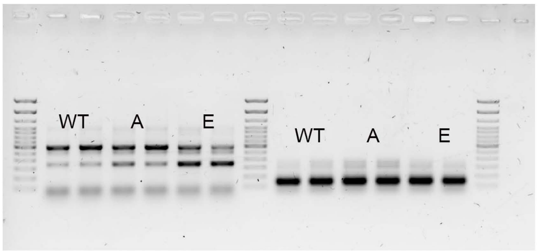

Oirignal images for panel (e)

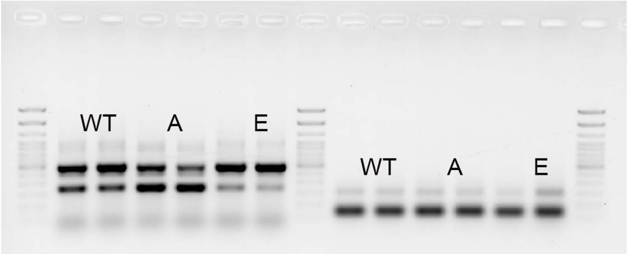

Supplement: Supplementary file 1 [file ijms-27-03094-s001.zip › ijms-4164889-supplementary.pdf]
